# High-flow nasal cannula therapy: clinical practice in intensive care units

**DOI:** 10.1186/s13613-019-0569-9

**Published:** 2019-09-04

**Authors:** Emmanuel Besnier, Sinad Hobeika, Saad NSeir, Fabien Lambiotte, Damien Du Cheyron, Bertrand Sauneuf, Benoit Misset, Fabienne Tamion, Guillaume Schnell, Jack Richecoeur, Julien Maizel, Christophe Girault, for the BoReal study group, G. Alvado, C. Andrejak, G. Barjon, C. Boulle, O. Delastre, P. Dubosq, H. Dupont, J. Elbaaj, J. C. Guilbaud, M. Hajjar, F. Hadjslimane, P. Hazera, P. Herbecq, O. Leroy, P. Jeanjean, M. Moubarak, S. Moulront, R. Pordes, E. Renaud, J. Rigaud, A. Riviere, D. Samiec, H. Shetiwy, D. Thevenin, T. Vanderlinden, C. Vinsonneau

**Affiliations:** 1 Department of Anaesthesiology and Critical Care, Rouen University Hospital, Rouen, France; 2 Inserm U1096 EnVi, Normandie Univ, Unirouen, Rouen, France; 3 Department of Medical Intensive Care, Rouen University Hospital, Rouen, France; 4 Department of Medical Intensive Care, Lille University Hospital, Lille, France; 5 Intensive Care Unit, Valenciennes Hospital, Valenciennes, France; 6 Department of Medical Intensive Care, Caen University Hospital, Caen, France; 7 Intensive Care Unit, Cherbourg-En-Cotentin Hospital, Cherbourg-En-Cotentin, France; 8 Intensive Care Unit, Le Havre Hospital, Le Havre, France; 9 Intensive Care Unit, Beauvais Hospital, Beauvais, France; 10 Department of Medical Intensive Care, Amiens University Hospital, Amiens, France; 11 Normandie Univ, Unirouen, UPRES EA-3830, Rouen, France

**Keywords:** Oxygen therapy, High-flow nasal cannula, Acute respiratory failure, Intensive care unit, Clinical practices

## Abstract

**Background:**

Despite the extensive use of high-flow nasal cannula (HFNC) therapy in intensive care units (ICU) for acute respiratory failure (ARF), its daily clinical practice has not been assessed. We designed a regional survey in ICUs in North-west France to evaluate ICU physicians’ clinical practice with HFNC.

**Materials and methods:**

We sent an observational survey to ICU physicians from 34 French ICUs over a 6-month period in 2016–2017. The survey included questions regarding the indications and expected efficiency of HFNC, practical aspects of use (initiation, weaning) and satisfaction. Comparisons between junior and senior ICU physicians were performed using a Fischer exact test.

**Results:**

Among the 235 ICU physicians contacted, 137 responded (58.3%) all of whom regularly used HFNC. Hypoxemic ARF was considered a good indication for HFNC by all 137, but only 30% expected HFNC success (i.e., avoiding intubation in at least 60% of cases). Among hypoxemic indications, 30% of juniors considered acute pulmonary edema a good indication versus 74% of seniors (*p* < 0.0001). Hypercapnic ARF was considered a good indication by 33% with only 2% expecting HFNC success. A need for conventional oxygen therapy ≥ 6 L/min justified HFNC therapy for 40% and ≥ 9 L/min for 39% of responders. 58% of ICU physicians started HFNC therapy with a FiO_2_ ≥ 50% and 28% with a gas flow ≥ 50 L/min. Practices for HFNC weaning were heterogeneous: 48% considered a FiO_2_ ≤ 30%; whereas, 30% considered a FiO_2_ ≤ 30% with a high flow ≤ 20 L/min. Criteria for HFNC failure (i.e., need for intubation) were ventilatory pauses or arrest (97%), persistent hypoxemia (95%), respiratory acidosis (81%), worsening of breathing (95%, 100% of seniors and 86% of juniors, *p* = 0.003), bronchial congestion (75%) and circulatory failure (61%, 72% of seniors and 44% of juniors, *p* = 0.007).

**Conclusion:**

HFNC is used by ICU physicians in many situations of ARF, despite their relatively low expectations of success, especially in cases of hypercapnia. Clinical practices appear somewhat heterogeneous. Despite the physiological benefit of HFNC, further prospective observational studies are still required on HFNC outcomes and daily practices.

## Introduction

High-flow nasal cannula (HFNC) is an oxygen support device recently developed as an alternative to conventional oxygen therapy (COT). HFNC consists of an air/oxygen blender connected through an active heated humidifier to nasal cannula. It allows adjustment of the fraction of inspired oxygen (FiO_2_) independent of the flow rate and the gas mixture. HFNC is associated with several physiological benefits and many studies have shown improvement in comfort and outcomes in various clinical settings [1]. Indeed, HFNC has been shown to be potentially useful and efficient in hypoxemic acute respiratory failure (ARF) [2], major post-operative care [3–5], immunocompromised patients [6, 7], for preoxygenation [8–11] or during bronchoscopy [12, 13]. Despite extensive literature exploring the interest of HFNC in critical care settings, very few studies, to our knowledge, have evaluated intensive care unit (ICU) physicians’ experience and their daily practice with HFNC in terms of clinical indications and modalities of use, as well as their subjective perception and confidence in the device. The objective of this study was, therefore, to evaluate ICU physicians’ daily clinical practice of the use of HFNC in ICUs in North-west France, as well as their perception of the usefulness of the device in various clinical settings.

## Materials and methods

### Study design

We conducted a prospective declarative survey during a 6-month period between October 2016 and March 2017. ICU physicians’ from The BoReal ICU study group (a clinical research network including 34 ICUs in North west France, 7 from University Hospital and 27 from General non University Hospital), were surveyed through a questionnaire sent electronically via the online software SurveyMonkey^®^ (https://fr.surveymonkey.com), developed according to available guidelines [14]. Briefly, the survey was designed by two ICU physicians (one senior and one junior) and then tested by five experienced ICU physicians before being sent to the whole population of interest. It was first sent in October 2016. Non-responders were contacted a second time in March 2017. One month later, ICU physicians who had not completed the survey were definitively categorized as non-responders. The survey was anonymous and responders were able to skip some questions at their discretion.

### Description of the survey

The complete survey is available in the Additional file 1. The first part of the survey was intended for the ICU Medical Head and included questions regarding demographic data and activity of the ICU during the year 2015 (number of ICU physicians, seniority, number of beds, number of admissions, use of mechanical ventilation). The second part was intended for all physicians of the ICU and divided into several sections: (1) usual indications of HFNC according to the ICU physician; (2) expected success of HFNC according to the ICU physician i.e. avoiding the need for intubation; (3) daily management of HFNC: criteria for initiation, initial parameters of use, modalities of use and HFNC weaning; (4) criteria of HFNC failure (need for intubation); and (5) global satisfaction. A psychometric Likert scale was used to assess the ICU physician’s perception for the different possible HFNC indications. Four proposals were suggested: “do not agree at all”, “rather do not agree”, “rather agree”, “totally agree”. The indication was classified as relevant if the ICU physician answered “rather agree” or “totally agree” and not relevant in the other cases. Regarding the expected rate of success of HFNC (i.e., avoiding intubation) according to the different indications, 6 propositions were possible: “not used in this indication”, “1–19%”, “20–39%”, “40–59%”, “60–79%”, “80–100%”. Responders were categorized as “juniors” for ICU physicians with less than 5 years, and “seniors” for those with more than 5 years of ICU experience.

### Statistics

Demographic data are presented as absolute number and median with first and third quartiles. Utilizations of HFNC, non-invasive ventilation (NIV) and invasive mechanical ventilation for 1000 admissions were calculated. Results concerning HFNC daily practice are presented as percentages and proportions of responses. Comparisons between the two groups (“juniors” vs “seniors”) were performed using a Fischer’s exact test or a χ^2^ test as needed. A p value < 0.05 was considered as statistically significant. All data were analyzed with Prism 6.0 (GraphPad, USA).

## Results

### Demographic data

All 34 ICU Medical Heads (from 7 university hospitals, 26 general hospitals and 1 private hospital) answered the demographic data of the survey. HFNC was available and regularly used in all ICUs. The median number of physicians in each medical team was 5.75 [5, 6] ICU physicians and 4 [3–6] residents. The median number of beds was 10.5 [8–12] for ICU and 5.5 [4–6.5] for intermediate care with an ICU physician for 2 [1.7–2.4] beds and a nurse for 2.6 [2.5–3] beds. During the year 2015, 17,134 patients were admitted to the 34 ICUs of the BoReal network, with a median of 798 [449–966] patients per ICU. A total of 10,202 patients were admitted for ARF with a median of 214 [189.5–390.3] patients per ICU, representing 59.5% of all admissions. During 2015, 2839 patients were treated with HFNC (166/1000 admissions), 2971 with NIV (173/1000) and 6604 with invasive mechanical ventilation (385/1000).

Regarding the second part of the survey, among the 235 ICU physicians contacted, 137 responded, i.e,. a response rate of 58.3%, with a median ICU expertise of 9 [5–18] years and a median HFNC use of 5 [3.5–7] years. All regularly used HFNC for ARF management (100%, 137/137). Of these responders, 20 did not answer to the items concerning indications or usual practice of HFNC, 6 answered less than 50% and 111 more than 50% of the items, resulting in a mean completion of the survey of 80.3% [79.6–81%].

### Indications for HFNC

Hypoxemic ARF was considered a relevant indication for HFNC by 100% of responders, and pneumonia (98%) and thoracic trauma (91%) were the preferred etiologies, followed by pulmonary embolism (85%), acute respiratory distress syndrome (ARDS) (71%), acute pulmonary edema (57%) and acute severe asthma (40%) (Table 1) 0.30% of responders expected HFNC therapy to be successful in avoiding intubation in at least 60% of hypoxemic ARF cases (Table 2). In contrast, 33% of responders considered hypercapnic ARF a relevant indication for HFNC. The main hypercapnic ARF etiologies were bronchial dilatations (32%), thoracic wall deformity (32%), chronic obstructive pulmonary disease (COPD) exacerbation (28%) and acute pulmonary edema (25%) (Table 1). Only, 2% of responders expected HFNC therapy to be successful in avoiding intubation in at least 60% of hypercapnic ARF cases (Table 2), which was significantly lower than for hypoxemic ARF (*p* < 0.0001). Nevertheless, 46% of responders estimated that HFNC had a 20–39% rate of success in hypercapnic ARF cases. The other potential indications considered for HFNC were post-extubation ARF prevention (44%), post-extubation ARF treatment (70%), post-operative ARF (76%), ARF for “ not to be resuscitated” patients (no intubation) for ethical reasons (92%), preoxygenation before endotracheal intubation (84%), and oxygenation during bronchoscopy (92%) (Table 1).

**Table 1:** Proportion of ICU physicians estimating the different potential indications of high-flow nasal cannula therapy as “good” or “very good”

	Overall, % (*n*)	Seniors, % (*n*)	Juniors, % (*n*)	p*
Hypoxemic ARF	100 (111/111)	100 (68/68)	100 (43/43)	1
Pneumonia	98 (109/111)	97 (66/68)	100 (43/43)	0.52
Thoracic trauma	91 (100/110)	90 (60/67)	93 (40/43)	0.74
Pulmonary embolism	85 (94/110)	87 (58/67)	84 (36/43)	0.78
ARDS	71 (78/110)	67 (45/67)	77 (33/43)	0.39
Acute pulmonary edema	57 (63/111)	74 (50/68)	30 (13/43)	< 0.0001
Acute severe asthma	40 (44/109)	45 (30/67)	33 (14/42)	0.32
“Do not intubate” patients	92 (100/109)	90 (60/67)	95 (40/42)	0.48
Per bronchoscopy	92 (97/106)	91 (58/64)	93 (39/42)	1
Preoxygenation before ETI	84 (86/102)	81 (51/63)	90 (35/39)	0.28
Post-operative ARF	76 (80/105)	77 (50/65)	75 (30/40)	0.82
Post-extubation ARF treatment	70 (74/105)	70 (45/64)	71 (29/41)	1
Post-extubation ARF prevention	44 (39/89)	45 (25/56)	42 (14/33)	1
Hypercapnic ARF	33 (27/83)	29 (15/52)	39 (12/31)	0.47
Bronchial dilatation	32 (35/108)	27 (18/67)	41 (17/41)	0.14
Thoracic wall deformity	32 (35/111)	30 (19/68)	37 (16/43)	0.40
COPD exacerbation	28 (31/110)	22 (15/67)	37 (16/43)	0.13
Acute pulmonary edema	25 (28/111)	31 (21/68)	16 (7/43)	0.12
Neuromuscular disease	20 (22/111)	19 (13/68)	21 (9/43)	0.81
Obesity hypoventilation syndrome	19 (21/111)	16 (11/68)	23 (10/43)	0.46
Acute severe asthma	14 (15/111)	15 (10/68)	12 (5/43)	0.78
Obstructive sleep apnea syndrome	7 (8/110)	6 (4/67)	9 (4/43)	0.71

ARF: Acute respiratory failure; ARDS: Acute respiratory distress syndrome; COPD: chronic obstructive pulmonary disease; ETI: endotracheal intubation. HFNC: high-flow nasal cannula; ICU: intensive care unit

*Comparisons were performed between junior and senior ICU physicians

**Table 2:** Expected success rates of high-flow nasal cannula therapy in the different indications

	Overall, % (*n*)	Seniors, % (*n*)	Juniors, % (*n*)	p*
All indications				0.95
1–19%	5 (5/104)	5 (3/66)	5 (2/38)	
20–39%	35 (36/104)	30 (20/66)	42 (16/38)	
40–59%	41 (43/104)	44 (29/66)	36 (14/38)	
≥ 60%	19 (20/104)	21 (14/66)	16 (6/38)	
Hypoxemic ARF				0.81
1–19%	6 (7/120)	5 (4/75)	7 (3/45)	
20–39%	23 (28/120)	19 (14/75)	31 (14/45)	
40–59%	41 (49/120)	45 (34/75)	33 (15/45)	
≥ 60%	30 (36/120)	31 (23/75)	29 (13/45)	
Hypercapnic ARF				0.88
1–19%	35 (16/46)	33 (9/27)	37 (7/19)	
20–39%	46 (21/46)	44 (12/27)	47 (9/19)	
40–59%	17 (8/46)	22 (8/27)	11 (2/19)	
≥ 60%	2 (1/46)	0	5 (1/19)	

ARF: Acute respiratory failure

*Comparisons were performed between junior and senior ICU physicians

### Practice of HFNC

Among patients treated for ARF, 40% of ICU physicians estimated that HFNC could be indicated for a minimal COT gas flow of 6 L/min, 39% for 9 L/min, 12% for 12 L/min and 9% for 15 L/min (Table 3). Initial settings varied according to ICU physician, with 58% starting HFNC with a 100% FiO_2_ and a gradual increase in gas flow and 28% with an initial gas flow ≥ 50 L/min. Regarding modalities for administration, 93% regularly used HFNC continuously and 54% regularly used it intermittently with NIV.

**Table 3:** Practices of high-flow nasal cannula therapy among ICU physicians (initiation, weaning, failure)

	Overall, *n* (%)	Seniors, *n* (%)	Juniors, *n* (%)	p*
Minimal COT gas flow justifying a switch to HFNC				0.67
6 L/min	40 (41/102)	38 (24/63)	44 (17/39)	
9 L/min	39 (40/102)	43 (27/63)	33 (13/39)	
12 L/min	12 (12/102)	10 (6/63)	15 (6/39)	
15 L/min	9 (9/102)	10 (6/63)	8 (3/39)	
Initial HFNC settings				
FiO_2_ ≥ 50%	58 (62/106)	53 (35/66)	68 (27/40)	0.16
Gas flow ≥ 50 L/min	28 (30/106)	33 (22/66)	20 (8/40)	0.18
Criteria for HFNC failure				
Breathing arrest	97 (103/106)	95 (40/42)	98 (63/64)	0.56
Refractory hypoxemia	95 (104/110)	94 (63/67)	95 (41/43)	1
Acidosis	81 (89/110)	78 (52/67)	86 (37/43)	0.33
Worsening of ARF	95 (104/110)	100 (67/67)	86 (37/43)	0.003
Bronchial congestion	75 (83/110)	82 (55/67)	72 (31/43)	0.24
Circulatory insufficiency	61 (65/106)	72 (48/67)	44 (17/39)	0.007
Agitation	95 (105/110)	99 (66/67)	91 (39/43)	0.08
Consciousness disorders	99 (109/110)	99 (66/67)	100 (43/43)	1
Other organ dysfunction	65 (71/109)	76 (50/66)	49 (21/43)	0.007
Criteria for HFNC weaning				0.33
FiO_2_ < 30%	50 (56/111)	57 (39/68)	40 (17/43)	
Gas flow < 20 L/min	16 (18/111)	9 (6/68)	12 (5/43)	
Both previous criteria	30 (33/111)	25 (17/68)	37 (16/43)	
Other	16 (18/111)	9 (6/68)	12 (5/43)	

ARF: Acute respiratory failure; COT: conventional oxygen therapy; HFNC: high-flow nasal cannula

*Comparisons were performed between junior and senior ICU physicians

Criteria for HFNC failure (i.e., need for intubation) were homogeneous with 95% of responders retaining the absence of correction of hypoxemia, 95% a worsening of ARF (increase in respiratory rate, nasal flaring, intercostal indrawing, suprasternal or supraclavicular retraction, and/or thoraco-abdominal paradoxical motion), 97% the occurrence of respiratory pauses or arrest, 81% the occurrence of acidosis, 75% the occurrence of a bronchial congestion and 61% circulatory insufficiency.

When respiratory conditions had improved, there was little variation in modalities for HFNC weaning among ICU physicians. Indeed, 81% declared that FiO_2_ should be reduced first, 6% that gas flow should be reduced first and 13% that both parameters should be reduced simultaneously. In contrast, there was more variation in the criteria for definitively stopping HFNC; the main criteria were a FiO_2_ below 30% for 50% of ICU physicians, a gas flow below 20 L/min for 16% and both criteria for 30% (Table 3).

### Comparison between “senior” and “junior” ICU physicians

No difference was observed between senior and junior ICU physicians regarding HFNC indications except for acute pulmonary edema responsible for hypoxemic ARF (Table 1). Indeed, 74% of seniors considered this indication of HFNC as relevant versus 30% of juniors (*p* < 0.0001). Moreover, no difference was observed for the expected success rates of HFNC in the various clinical situations (Table 2).

Similarly, there was no difference for COT gas flow justifying a switch to HFNC therapy or for the initial HFNC settings (Table 3). On the other hand, differences were found for criteria of HFNC failure. If 100% of seniors considered that worsening of ARF during HFNC therapy required endotracheal intubation, only 86% of juniors did (*p* = 0.003). Circulatory insufficiency was also considered as a cause for endotracheal intubation for 72% of seniors versus only 44% of juniors (*p* = 0.007), as well as a new organ dysfunction for 76% of seniors versus only 49% of juniors (*p* = 0.007). No difference was observed between the two groups for the modalities of HFNC weaning (Table 3).

The overall satisfaction of HFNC therapy was estimated as excellent and satisfactory in, respectively, 85% (93/110) and 15% (17/100) of responders, with no difference observed between juniors and seniors (84% (36/43) versus 85% (57/67) and 16% (7/43) versus 15% (10/67), respectively; *p* = 1).

## Discussion

Our study evaluates the clinical experience and perception of ICU physicians in their use of HFNC therapy. This regional multicenter survey shows that ICU physicians in Northwest France use HFNC therapy in their daily practice in a wide range of indications, but mainly in hypoxemic ARF. The survey also highlights some differences in the practical use of HFNC between senior and junior ICU physicians.

### Indications for HFNC

Not surprisingly, hypoxemic ARF is considered by all ICU physicians in our survey as a good indication for HFNC, with pneumonia as preferred etiologies. Indeed in the randomized controlled study FLORALI including 310 patients, more than 80% of the ARF population had pneumonia [2], providing a good level of evidence in this indication. Interestingly and despite the lack of literature evidence, 85% of practitioners believe that HFNC could be used in pulmonary embolism. Indeed, only one retrospective study reported an improvement in oxygenation and respiratory rate in 17 cases of pulmonary embolism treated with HFNC [15]. Prospective clinical trials are, therefore, still needed to assess the efficiency and safety of the device in this situation. From a physiological point of view, however, the high flow of oxygen could probably be able to counteract the alteration of ventilation–perfusion ratios without having a major negative impact on right ventricular function. Concerning HFNC in cardiogenic pulmonary edema, there were no specific clinical data supporting its use at the time of the survey, despite HFNC can generate low levels of positive inspiratory and expiratory pressures [16]. However, the levels generated remain much lower than those with NIV. Nevertheless, the combination of NIV and HFNC could probably be useful in this indication [17]. It was striking to note that only 30% of ICU physicians in our study expect HFNC treatment to be successful in more than 60% of cases of hypoxemic ARF, while the FLORALI study observed an actual success rate of 62% [2]. The relatively low rate of expected HFNC success in hypoxemic ARF in our survey could reflect the few evidences regarding the potential benefit of HFNC in hypoxemic ARF at the date of the survey or the fact that some physicians could not have been already aware of the results. Indeed, apart from the FLORALI study [2], only few observational trials have suggested a potential favorable outcome with HFNC support in hypoxemic ARF, with similar level of HFNC success (65%) [18]. Moreover, data have suggested that high tidal volumes can be responsible for Volume Induced Lung Injuries (VILI) [19], even during NIV therapy [20], which in association with an excess in respiratory drive in patients with spontaneous breathing could promote Patient Self-Induced Lung Injury (P-SILI) in hypoxemic ARF [21]. This unexpected increase in lung volume has recently been demonstrated with NIV [22] which could explain the risk of failure and poor outcome with this ventilatory support in hypoxemic ARF [2, 22]. Finally, based on the current literature, the physiological benefits of HFNC and the potential risk of P-SILI with NIV, it seems reasonable to favor HFNC as the first-line therapy in hypoxemic ARF [23].

Regarding hypercapnic ARF, only one-third of physicians in our survey consider this indication as potentially relevant. This could reflect the lack of reliable clinical evidence in this field. Although the physiological effects of HFNC, mainly by decreasing CO_2_ re-breathing due to the anatomical dead space washout, were demonstrated to be potentially useful in stable COPD [24] and to limit or decrease acute hypercapnia [25], no large randomized prospective clinical study has been performed in hypercapnic ARF as yet. Some case reports have suggested a beneficial effect of HFNC to manage hypercapnic ARF in COPD patients unable to tolerate NIV [26]. Other recent studies published after our survey suggest clinical benefits of HFNC in cases of hypercapnia [27–29]. Up to now, only one small prospective randomized study has compared NIV and HFNC in 88 severe COPD exacerbations [29]. However, no difference was reported on clinical outcome including 30-day intubation and survival. The lack of clinically relevant studies could explain, therefore, the low success rate expected with HFNC support in hypercapnic ARF (less than 2% of responders expected a success rate of more than 60%). Nevertheless, 46% of ICU physicians estimate that the success rate could be between 20 and 40%, suggesting this technique could be considered as a reliable alternative to NIV in intolerant hypercapnic ARF patients or a complementary support in most severe situations. Further large clinical trials are needed to support this indication. In this context, the results of the French prospective randomized multicenter “High-Flow ACRF” trial will be helpful [30].

For other indications, only 44% of physicians in our survey consider that HFNC is potentially relevant to prevent post-extubation ARF. Previous studies have suggested some physiological benefits of HFNC during the post-extubation period [31, 32]. Two multicenter prospective randomized trials have demonstrated that HFNC could be better than COT [33] and not inferior to NIV [34] to prevent reintubation and post-extubation ARF in low- and high-risk patients, respectively. Results of the ongoing “High-Wean” trial comparing HFNC alone or with NIV in the post-extubation period for high-risk patients should give further evidence in this indication [35].

For patients with “do not intubate” instructions, more than 90% of ICU physicians in our survey consider that HFNC is a useful respiratory support. This application was later evaluated as beneficial in a retrospective study including 84 patients treated with NIV or HFNC showing a similar poor survival rate, but a longer maintenance of the ability to speak or eat for patients with HFNC [36]. HFNC can, thus, combine respiratory comfort, high satisfaction and potential benefit on outcome with a possible recovery for these patients that could make HFNC a first-line respiratory support in these difficult situations.

More than 80% of ICU physicians in our survey consider that HFNC is useful as a preoxygenation technique before endotracheal intubation. Interestingly, data available at the time of this survey were limited to two studies. An observational before–after study suggested that HFNC can significantly improve preoxygenation and reduce severe intubation-related hypoxemia in comparison with non-rebreathing facemask [11]. The other, a multicenter randomized controlled trial, failed to show any difference between these two preoxygenation strategies [8]. Several studies have since been published [10, 37–39]. None of them has provided unquestionable and definite data as to the best strategy to preoxygenate ICU patients.

Finally and surprisingly, results of our survey highlight that despite the lack of strong evidence-based data in the different indications identified as potentially relevant, HFNC can be readily and widely accepted and used by ICU physicians. This is quite unique in the history of ICU techniques and management, and clinicians should be careful in poorly studied indications, although the physiological benefit and simplicity of HFNC devices could explain the wide interest for this technique [1].

### Practical modalities of HFNC use

More than half of the ICU physicians in our survey use a high FiO_2_ level at HFNC initiation, above 50%. This is in accordance with some clinical study protocols that recommend the use of FiO2 up to 50 or 100% [2, 4]. Surprisingly, only 30% use a high-flow rate (at least 50 L/min) at HFNC initiation in contrast with most randomized clinical trials [4, 17, 31, 40]. Indeed, a large part of the physiological benefits lies in the HFNC flow rate applied, which can proportionally reduce respiratory work, improve oxygenation, compliance and pulmonary aeration, independent of FiO_2_ [41]. In the same way, more than half of the ICU physicians in our study indicate that a low FiO_2_ was the main criterion for HFNC weaning, without considering the flow rate. For the same reasons mentioned above, the HFNC flow rate should be considered in the weaning process. To our knowledge, no work has specifically explored the different modalities of HFNC weaning in daily practice.

### Comparisons between senior and junior ICU physicians

Very few differences are found between senior and junior ICU physicians in the practice of HFNC. Seniors are more likely to consider HFNC in hypoxemic cardiogenic pulmonary edema as compared to juniors without clear explanation or evidence-based data for this finding. In addition, seniors most often consider a circulatory, respiratory or other organ failure as a criterion for HFNC failure. These small differences could be due to a greater confidence in the technology use for juniors. Indeed, HFNC has been widely used in our region during the last decade. Thus, most young ICU physicians have known this technique since the beginning of their practice, increasing probably their confidence in the device.

### Strengths and limits

This study focuses on ICU physicians’ expectations and their self-reported practice regarding HFNC, beyond any clinical trials. It particularly highlights the discrepancy between the lack of evidence for numerous potential indications at the time of the survey and the wide use of HFNC in clinical practice. Some limitations should also be underlined. First, the survey was not designed according to the Delphi method. Nevertheless, to limit the potential bias related to the methodology, we followed the available recommendations for self-administered questionnaire [14]. Second, a possible discrepancy between physicians’ responses and their actual practices cannot be formally excluded. Third, the possibility of skipping questions could also lead to bias in data collection, although it guaranteed a higher response rate. The 58% response rate could be considered as relatively low, but a rate between 50 and 60% is often considered acceptable and reported in the literature for this type of study [42]. Fourth, this survey has been performed more than 2 years ago and could not represent the current practices with HFNC in ICU because of the growing literature in this field. In addition, some of the results, notably those concerning the indications of HFNC, could reflect the lack of knowledge of the ICU physicians rather than conflicting results from the literature. Finally, although the survey was conducted with the French BoReal research network not specifically dedicated to respiratory support, results may not be representative of ICU physicians’ clinical experience and perception with HFNC across the France or around the world.

## Conclusion

In conclusion, HFNC therapy is used by ICU physicians in the North-west region of France in many situations of ARF despite limited available evidence in most of these indications at the time of the survey. While criteria for HFNC failure were found homogeneous, there were some discrepancies between ICU physicians regarding the strategies and criteria for HFNC weaning. Despite the physiological benefit and simplicity of HFNC technique, further prospective studies on clinical practices and relevant outcomes with HFNC are still warranted.

## Supplementary information

**Additional file 1.** Survey on the daily practice of high-flow nasal cannula therapy.

## Data Availability

The datasets used and/or analyzed during the current study are available from the corresponding authors on reasonable request

## Abbreviations

ARDS: acute respiratory distress syndrome
ARF: acute respiratory failure
COPD: chronic obstructive pulmonary disease
COT: conventional oxygen therapy
FiO_2_: fraction of inspired oxygen
HFNC: high-flow nasal cannula
ICU: intensive care unit
NIV: non-invasive ventilation
