# Supplementary material for: High-flow nasal cannula therapy: clinical practice in intensive care units
Source: Ann Intensive Care. 2019 Sep 4;9:98. doi: 10.1186/s13613-019-0569-9 (PMC6726730; doi:10.1186/s13613-019-0569-9)
Supplement: Supplementary file 1 — Additional file 1. Survey on the daily practice of high-flow nasal cannula therapy. [file 13613_2019_569_MOESM1_ESM.doc]

Survey on the daily practice of High-Flow Nasal Cannula therapy in the French BoReal study network

_1_ **We do not practice HFNC in our service for the management of Acute Respiratory Failure (ARF):**

Please explain why ? : ………………………………………………………………………………………………………………………

………………………………………………………………………………………………………………………

………………………………………………………………………………………………………………………

_2_ **We do practice HFNC in our service for the management of Acute Respiratory Failure (ARF)**

**Intensivist**

First name : ……………………………………………………………………………………………

last name  : ……………………………………………………………………………………….

position :

1 Professor

2 assistant professor

3 phycisian

4 resident chief

5 other: …………………………………………………………………………..

expertise in Intensive care unit (ICU) in years : |__|__|

**d**ate (dd/mm/yyyy) : |__|__|-|__|__|-|__|__|__|__|

**ICU**

HOSPITAL : 1UNIVERSITY HOSPITAL

2GENERAL HOSPITAL

3OTHER: …………………………………………………………………..

SERVICE : 1 MEDICAL ICU

2 SURGICAL ICU

3 MIXED ICU

5 CARDIAC ICU

6 NEUROSURGICAL ICU

7 RESPIRATORY ICU

8 INTERMEDIATE CARE UNIT

9 OTHER : ……………………………………………………………………

**ADRESS**: ………………………………………………………………………………………………………….

………………………………………………………………………………………………………….

**e**-mail: …………………………………………………………………………………………

**1. CHARACTERISTICS OF THE ICU (To fill only by the manager of the ICU)**

- beds : |__|__|
  - ICU : |__|__|
  - Intermediate care :
- Intensivists : |__|__|
- Residents : |__|__|
- Physiotherapists : |__|__|
- Nurses : |__|__|

**2. ARF MANAGEMENT IN YOUR ICU OVER THE 2015 PERIOD**

**(TO FILL ONLY BY THE MANAGER OF THE ICU)**

**2.1** Number of admissions in your ICU : |__|__|__|__|

**2.2** Number of admissions for ARF : |__|__|__|

**2.3** Number of patients treated with conventional oxygen therapy (COT) |__|__|__|

**2.4** Number of patients treated with no invasive ventilation (NIV) |__|__|__|

**2.5** Number of patients treated with invasive mechanical ventilation |__|__|__|

**2.6** Number of patients treated with HFNC |__|__|__|

**3. Efficiency of HFNC in your unit**

**3.1According to you, what is the expected rate of success of HFNC (*i.e.* avoiding intibation) in your unit:**

NU 1-19 20- 39 40-59 60-79 80-100%

- All indications : _0_ _1_ _2_ _3_ _4_  _5_
- Hypoxemic ARF: _0_ _1_ _2_ _3_ _4_ _5_
- Hypercapnic ARF: _0_ _1_ _2_ _3_ _4_ _5_
- Post-extubation: _0_ _1_ _2_ _3_ _4_  _5_
- Other: _0_ _1_ _2_ _3_ _4_  _5_

Please specify : ………………………………………………………………………………………………….

* NU = Not used in this indication

**4. Expertise in HFNC therapy**

**4.1 For how long do you practice HFNC (in years)?** |__|__|__|__|

**5. In which indications do you use Hfnc?**

***Do not agree Rather do Rather Totally***

***at all not agree agree agree***

**5.1** _1_ **Hypoxemic ARF :** _0_ _1_ _2_ _3_

- Acute pulmonary oedema: _0_ _1_ _2_ _3_
- Severe asthma:  _0_ _1_ _2_ _3_
- Pneumonia : _0_ _1_ _2_ _3_
- ARDS : _0_ _1_ _2_ _3_
- Pulmonary embolism : _0_ _1_ _2_ _3_
- Thoracic trauma : _0_ _1_ _2_ _3_
- Others: _0_ _1_ _2_ _3_

Please specify : ………………………………………………………..

**5.2** _2_ **Hypercapnic ARF :** _0_ _1_ _2_ _3_

- COPD : _0_ _1_ _2_ _3_
- Bronchus dilatation: _0_ _1_ _2_ _3_
- Hypercapnic pulmonary oedema: _0_ _1_ _2_ _3_
- Hypercapnic severe asthma: _0_ _1_ _2_ _3_
- Obesity-hypoventilation syndrome: _0_ _1_ _2_ _3_
- Sleep Apnoea syndrome : _0_ _1_ _2_ _3_
- Thorac wall deformity : _0_ _1_ _2_ _3_
- Pathologies neuromusculaires : _0_ _1_ _2_ _3_
- Others : _0_ _1_ _2_ _3_

Please specify: ………………………………………………………

**5.3** _3_ **during Post-extubation period :**  _0_ _1_ _2_ _3_

**5.4** _4_ **post-extubation ARF** _0_ _1_ _2_ _3_

**5.5** _5_ **post-operative ARF :** _0_ _1_ _2_ _3_

**5.6** _6_ **Do not intubated patients (ethical issues) :** _0_ _1_ _2_ _3_

**5.7** _7_ **Invasive procedures :** _0_ _1_ _2_ _3_

préciser :

- Pre-oxygenation before intubation : _0_ _1_ _2_ _3_
- Bronchoscopy : _0_ _1_ _2_ _3_
- Others : _0_ _1_ _2_ _3_

Please specify : …………………………………………………………

**5.8** _8_ **Others indications**: _0_ _1_ _2_ _3_

Please specify: ……………………………………………………………………

**6. Practices concerning HFNC use**

**6.1** **Is a written protocol available in your unit ? : YES** _1_ **NO** _0_

**6.3** **Device(s) used** :

- Specific devices **YES** _1_ **NO** _0_
  - Optiflow® : **YES** _1_ **NO** _0_
  - Airvo® : **YES** _1_ **NO** _0_
  - Other : **YES** _1_ **NO** _0_

Please specify : …………………………………………………………

- ICU ventilator with HFNC function **YES** _1_ **NO** _0_

**6.4 Conventional Oxygen Flow requiered for switch to HFNC :**

- Whaterever the oxygen flow **YES** _1_ **NO** _0_
- Oxygen flow > 3L /mn **YES** _1_ **NO** _0_
- Oxygen flow > 6L /mn  **YES** _1_ **NO** _0_
- Oxygen flow > 9L /mn  **YES** _1_ **NO** _0_
- Oxygen flow > 12L /mn **YES** _1_ **NO** _0_
- Oxygen flow > 15L /mn  **YES** _1_ **NO** _0_

**6.5 Initial settings of HFNC :**

- FiO_2_ = 100% and gas flow > 50L/min **YES** _1_ **NO** _0_
- FiO_2_ = 50-100% and gas flow 30-50L/min **YES** _1_ **NO** _0_
- FiO_2_ = 50-100% and progressive rise in gas flow **YES** _1_ **NO** _0_
- Progressive rise in FiO_2_ and gas flow 30-50L/min  **YES** _1_ **NO** _0_
- Progressive rise in FiO_2_ and gas flow **YES** _1_ **NO** _0_
- Others : **YES** _1_ **NO** _0_

Please specify : ………………………………………………………………….

**6.6 Modalities of application :**

***Never Sometimes Often Always***

- Continuous _0_ _1_ _2_ _3_
- Sequential _0_ _1_ _2_ _3_
  - HFNC/COT : _0_ _1_ _2_ _3_
  - HFNC/NIV _0_ _1_ _2_ _3_
- Others _0_ _1_ _2_ _3_ Please specify : ………………………………………………………………….

**6.7** **Weaning from HFNC** :

- First reduce FiO_2_ **YES** _1_ **NO** _0_
- First reduce gas flow **YES** _1_ **NO** _0_
- Both **YES** _1_ **NO** _0_
- Others **YES** _1_ **NO** _0_

Please specify : ………………………………………………………………….

**6.8** **Criteria for stopping HFNC** :

- FiO_2_ < 30% **YES** _1_ **NO** _0_
- Gas flow < 20L/mn **YES** _1_ **NO** _0_
- Both criteria **YES** _1_ **NO** _0_
- Autre(s) critère(s) **YES** _1_ **NO** _0_

précisez : ………………………………………………………………….

**7. Criteria of HFNc failure : need for intubation ?**

***Do not agree Rather do Rather Totally***

***at all not agree agree agree***

- Pauses or stops in breathing _0_ _1_ _2_ _3_
- Refractory hypoxemia  _0_ _1_ _2_ _3_

(SpO2 ≤ 90¨% with FiO2 100¨%)

- Respiratory acidosis _0_ _1_ _2_ _3_
- Worsening of ARF _0_ _1_ _2_ _3_

(BR > 35/mn and/orPaO2/FiO_2_ < 120 mmHg)

- Bronchus congestion _0_ _1_ _2_ _3_
- Circulatory failure (SBP ≤ 90 mmHg) _0_ _1_ _2_ _3_
- Cardiac arrhythmia or atrioventricular blockade _0_ _1_ _2_ _3_
- Agitation/confusion _0_ _1_ _2_ _3_
- Disorders in consciousness _0_ _1_ _2_ _3_
- Other organ dysfunction _0_ _1_ _2_ _3_
- Other

Please specify :

. ………………………………….…………… _0_ _1_ _2_ _3_ ………………………………….…………… _0_ _1_ _2_ _3_

**8. GLOBAL satisfaction**

_1_ Very satisfied

_2_ Moderatly satisfied

_3_ not very satisfied

_4_ not satisfied at all

**8.2 If your are not satisfied, why ?**

_1_ Frequent unsuccess

_2_ Work overload

_3_ Lack of devices

_4_ Lack of motivation from the medical staff

_5_ Lack of motivation from the nurse staff

_6_ Lack of scientific evidence

_7_ Others :………………………………….…………………………………………….

**9. Comments**

.………………………………….…………………………………………………………………………………...

.………………………………….…………………………………………………………………………………...

.………………………………….…………………………………………………………………………………...

.………………………………….…………………………………………………………………………………...

.………………………………….…………………………………………………………………………………...
